# Persistent Post COVID-19 Endothelial Dysfunction and Oxidative Stress in Women

**DOI:** 10.3390/pathophysiology31030033

**Published:** 2024-08-28

**Authors:** Natalya Semenova, Ekaterina Vyrupaeva, Sergey Kolesnikov, Marina Darenskaya, Olga Nikitina, Lyubov Rychkova, Liubov Kolesnikova

**Affiliations:** Scientific Centre for Family Health and Human Reproduction Problems, Irkutsk 664003, Russia; goliafm@yandex.ru (E.V.); sikolesnikov2012@gmail.com (S.K.); marina_darenskaya@inbox.ru (M.D.); olga_tolpygina@mail.ru (O.N.); iphr@sbamsr.irk.ru (L.R.); kolesnikova20121@mail.ru (L.K.)

**Keywords:** endothelial dysfunction, oxidative stress, antioxidant status, COVID-19, post-COVID, menopause

## Abstract

The assessment of endothelial dysfunction and free radical homeostasis parameters were performed in 92 women, aged 45 to 69 years, divided into the following groups: women without COVID-19 (unvaccinated, no antibodies, control); women with acute phase of COVID-19 infection (main group, COVID-19+); 12 months post COVID-19+; women with anti-SARS-CoV-2 IgG with no symptoms of COVID-19 in the last 12 months (asymptomatic COVID-19). Compared to the control, patients of the main group had lower glutathione peroxidase (GPx) and superoxide dismutase (SOD) activities, decreased advanced glycation end products (AGEs) level, higher glutathione reductase (GR) activity, and higher glutathione S transferases pi (GSTpi), thiobarbituric acid reactants (TBARs), endothelin (END)-1, and END-2 concentrations (all *p* ≤ 0.05). The group with asymptomatic COVID-19 had lower 8-OHdG and oxidized glutathione (GSSG) levels, decreased total antioxidant status (TAS), and higher reduced glutathione (GSH) and GSH/GSSG levels (all *p* ≤ 0.05). In the group COVID-19+, as compared to the group without clinical symptoms, we detected lower GPx and SOD activities, decreased AGEs concentration, a higher TAS, and greater GR activity and GSTpi and TBARs concentrations (all *p* ≤ 0.05). The high content of lipid peroxidation products 12 months post COVID-19+, despite decrease in ENDs, indicates long-term changes in free radical homeostasis. These data indicate increased levels of lipid peroxidation production contribute, in part, to the development of free radical related pathologies including long-term post COVID syndrome.

## 1. Introduction

During menopause, women begin to experience age-related neuroendocrine changes, accompanied by estrogen deficiency, which is associated with greater vulnerability to moderate and severe COVID-19 and subsequent complications [1]. Complications may also develop from COVID-19 due to the formation of endothelial dysfunction in these patients [2,3,4,5,6]. It has been shown that, a year after infection, menopausal women continue to experience physical and emotional health problems associated with COVID-19, which may be due to endothelial changes [7,8]. One indicator of endothelial dysfunction is changes in the level of endothelin, which exists in three isoforms, each with a slightly different amino acid sequence and place of production in the body. Specifically, endothelin-1 (END-1) is produced by the cells of almost all organs, endothelin-2 (END-2) is produced by the intestines and kidneys, and endothelin-3 (END-3) is found in high concentrations in nervous tissue [9]. The few studies conducted on endothelial dysfunction in patients with COVID-19 have assessed only the END-1 levels, however, there is no consensus on how these levels differ depending on the severity of the disease [10,11].

One of the mechanisms for the development of endothelial dysfunction is oxidative stress, which develops during infection with the SARS-CoV-2 virus [3,12,13,14,15,16,17]. Under normal conditions, the free radical balance in the body is preserved by the antioxidant defense system (AOD), predominantly via the protein glutathione, which is involved in almost all stages of protection against oxidative stress [18]. The functions of glutathione can be realized through the appropriate enzymes, which expand its properties to enable the protection of macromolecules. Glutathione S transferase (GST) catalyzes the conjugation reactions of glutathione with nonpolar substrates. Several different classes of GSTs are known, but the pi class is the most commonly studied one due to its relationship with the development of human diseases [19,20]. Glutathione reductase (GR) is a reducing agent for oxidized glutathione (GSSG), and glutathione peroxidase (GPx) restores free hydrogen peroxide and hydroperoxides [21,22].

Superoxide dismutase (SOD) is a metal-containing enzyme that inactivates the superoxide anion radical and is an active participant in the first line of defense against free radicals. There are several isoforms of this enzyme, which differ in terms of their metal cofactors in the active center. These include copper–zinc superoxide dismutase (Cu/ZnSOD, SOD1), located in the cytoplasm; manganese superoxide dismutase (MnSOD, SOD2), located in the mitochondria; and extracellular superoxide dismutase (EcSOD, SOD3) [23]. Taking into account the different locations of these SOD isoforms, determining their overall activity is important for understanding the processes catalyzed by the enzyme throughout the body.

Though the increase in levels of reactive oxygen species during a COVID-19 infection is well-established, studies of the antioxidant status involving both the glutathione system and SOD activity during SARS-CoV-2 infection have produced ambiguous results [12,13,14,15,16,24,25,26], possibly due to a lack of control groups and consideration of age and gender, factors that may influence the activity of the AOD system [27,28,29].

Therefore, the aim of this investigation is to assess endothelin isoforms, oxidative stress, and antioxidant status in menopausal women with moderate COVID-19, in patients twelve months after contracting the disease, and in cases of asymptomatic COVID-19 to determine whether this cohort was more vulnerable to the risks associated with COVID-19 infection.

The hypothesis of this study is the development of oxidative stress and endothelial dysfunction in menopausal women with moderate COVID-19, which persists 1 year after infection, as well as the balance of free radical homeostasis in asymptomatic disease.

## 2. Materials and Methods

### 2.1. Standard Protocol Approvals, Registrations, and Patient Consent

We employed a case–control research design (to explore the differences between the control and COVID-19) and a retrospective cohort study design (to examine the acute-phase parameters and the post-COVID-19 parameters). The study was carried out in the Federal State Public Scientific Institution under the project “Scientific Centre for Family Health and Human Reproduction Problems”. Informed consent was given by the participants in accordance with the Ethical Norms of the Declaration of Helsinki of the World Medical Association (2013). The Research Protocol was approved by the Committee on Biomedical Ethics of the Scientific Centre (protocol No 6.1, dated 19 June 2020).

### 2.2. Subjects

**Figure 1:**
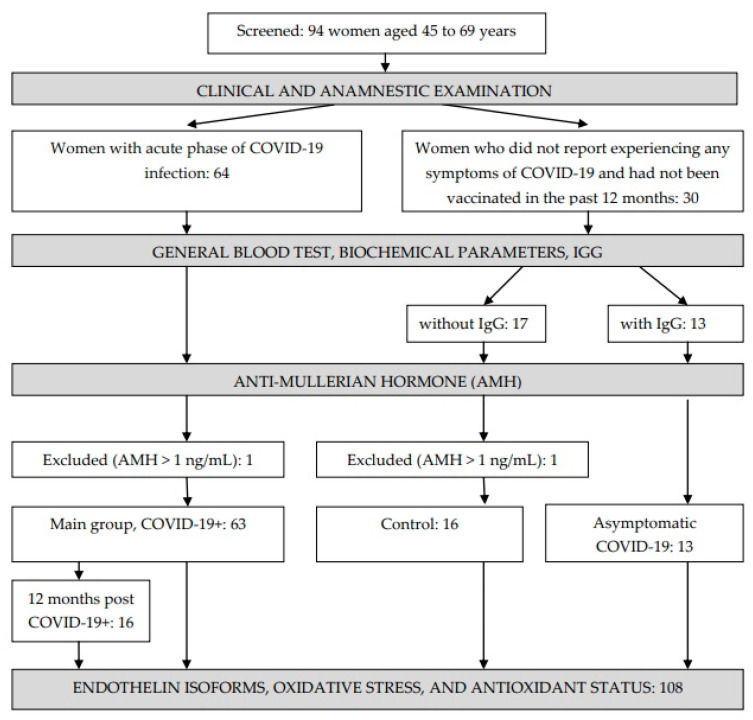
Study design.

The study included 94 women aged 45 to 69 years (Figure 1). Of these, 64 women were selected for inclusion in the main cohort (COVID-19+); these women were hospitalized in the Irkutsk Regional Infectious Clinical Hospital during the period from June 2020 to March 2021, had a laboratory-confirmed PCR test for the presence of the SARS-CoV-2, and displayed moderate COVID-19 symptoms accompanied by pneumonia. Upon the patients’ admission to the hospital, questionnaires, an analysis of medical records, a general clinical examination, and computed tomography were performed. One woman with an anti-Mullerian hormone (AMH) level of 5.18 ng/mL was excluded from the main group. The distribution of patients by the degree of lung damage according to the results of computed tomography was as follows: CT-1 (54%), CT-2 (33.3%), CT-3 (12.7%). Among those who survived COVID-19, 16 women who were called for a clinical and anamnestic examination agreed to be examined again after 12 months.

Thirty women who did not report experiencing any symptoms of COVID-19 and had not been vaccinated in the past 12 months were included in the control group. The presence of COVID-19 IgG antibodies in the blood was checked in all women, after which two groups were formed: those without IgG (*n* = 17) and those with IgG (*n* = 13). One woman with an AMH level of 14.61 ng/mL was excluded from the group without IgG; thus, 16 women formed the control group. A further 13 women with IgG in their blood formed a separate group: those with asymptomatic COVID-19.

All participants were examined by a general practitioner–cardiologist via calculation of their body mass index (BMI), measurement of their blood pressure and body temperature, and an electrocardiogram. In addition, all women noted the presence of amenorrhea or menstrual irregularities, consisting of stable fluctuations (7 days and above) during successive cycles. To exclude the possibility of COVID-19 being present at the time of the study (control, asymptomatic COVID-19, post-COVID-19), an appropriate rapid test (RAPID BIO, Ulan-Ude, Russia) was performed.

The exclusion criteria for all groups were as follows: a regular menstrual cycle, the use of hormone-replacement therapy, and/or an AMH level > 1 ng/mL (for all participants). The exclusion criteria for the control group were as follows: exacerbation of chronic diseases, the presence of IgG to SARS-CoV-2, a positive result for the presence of SARS-CoV-2. The only exclusion criterion for the main group was the absence of pneumonia.

### 2.3. Methods

#### 2.3.1. Collection of Materials

Between 8.00 and 9.00 a.m., after 12 h of overnight fasting, venous blood was sampled from the cubital vein into two tubes (with EDTA-K3 to obtain erythrocyte lysate and a clot activator to obtain serum). Whole venous blood was used immediately to carry out a full blood count. Then, the samples were centrifuged for 10 min at 1500× *g* and blood serum was used immediately for the determination of the aspartate aminotransferase (AST) and alanine aminotransferase (ALT) concentrations. The remaining blood serum was collected in an Eppendorf tube and frozen. The erythrocytes were washed three times with 0.9% NaCl and centrifuged for 5 min at 1500× *g* after each wash. Afterwards, erythrocytes were resuspended in bi-distilled water at a 1:2 ratio, incubated for 10 min at 2 to 8 °C, and then centrifuged at 1500× *g* for 5 min; the stroma was removed, and the final 100 µL lysate output was mixed with 1.9 mL 0.9% NaCl and frozen. Serum was used to assess the GR activity, TAS, AMH, and C-reactive protein (CRP), GSTpi, 8-OHdG, AGEs, and TBARs concentrations. Plasma was used to assess the AOPP and END-1, -2, -3 concentrations. Meanwhile, hemolysate was used to determine the GSH and GSSG levels and GPx and SOD activities. The samples were stored at −40 °C prior to the assays being carried out.

#### 2.3.2. General Blood Test, Biochemical and Hormonal Parameters, IgG

The full blood count parameters (erythrocytes, leukocytes, neutrophils, lymphocytes, monocytes, eosinophils, thrombocytes, and hemoglobin) were determined using a blood analysis machine (Mindray BC-5300, Shenzhen, China) with the appropriate reagents (Shenzhen Mindray Bio-Medical Electronics Co., Ltd., Shenzhen, China; 0054, 2356, 8396, 0201; Mindray Medical Rus distributor). AST and ALT were assayed using commercial kits (Vital, Saint Petersburg, Russia; B 02.16, B 01.16) on an automatic photometer (BTS-330, BioSystems, Barcelona, Spain). The AMH (ng/mL), CRP (pg/mL), and IgG (BAU/mL) levels were determined using a microplate reader (MultiSkan ELX808, Biotek, Winooski, VT, USA) with a test system (Hema, Balashikha, Russia; K245) and a commercial kit (Vector-Best, Novosibirsk, Russia; A-9002).

#### 2.3.3. Endothelin Isoforms

END-1, -2, and -3 concentrations were determined using commercial enzyme-linked immunosorbent assay kits (Cloud-Clone Corporation, Houston, TX, USA; CEA482Hu, CEF415Hu, CEC465Hu; ProteinsAntibodies RF distributor) on a microplate reader (MultiSkan ELX808, Biotek, Winooski, VT, USA) at λ = 450 nm. The END concentrations are expressed in pg/mL.

#### 2.3.4. Oxidative Damage Products

Plasma was analyzed to determine the levels of oxidative damage products (TBARs, AOPP, 8-OHdG, and AGEs).

Commercial kits (Agat, Moscow, Russia; 74/53) were used to detect the TBARs level. This method uses lipid peroxidation products, which form a colored complex with thiobarbituric acid (TBA), which can then be extracted with butanol. Plasma TBARs levels were determined using the TBA reaction, which was followed by the detection of the fluorescence intensity (at λ = 515 nm (excitation) and λ = 554 nm (emission)). The TBARs measurements were carried out with a spectrofluorophotometer (Shimadzu RF-1501, Tokyo, Japan) and are expressed in μmol/L.

The AGEs levels were determined using commercial enzyme-linked immunosorbent assay kits (Cloud-Clone Corporation, Houston, TX, USA; CEB353Ge; ProteinsAntibodies RF distributor) on a microplate reader (MultiSkan ELX808, Biotek, Winooski, VT, USA) at λ = 450 nm. The AGEs’ concentrations are expressed in ng/mL.

The 8-OHdG concentrations were determined using commercial enzyme-linked immunosorbent assay ELISA kits (Assay Design DNA Damage, Enzo LifeSciences Inc., Farmingdale, NY, USA; ADI-EKS-350; BioChemMack distributor, Moscow, Russia) on a microplate reader (MultiSkan ELX808, Biotek, Winooski, VT, USA) at λ = 450 nm. The kit is based on a fast and sensitive competitive enzyme immunoassay and is designed to determine 8-OHdG levels in urine, serum, and saliva samples. The 8-OHdG concentration is expressed in ng/mL.

The AOPP levels were determined via spectroscopic analysis (Immundiagnostik, Bensheim, Germany; KR7811w; BioChemMack distributor) on a microplate reader (MultiSkan ELX808, Biotek, Winooski, VT, USA) at λ = 340 nm. The AOPP concentrations are expressed in nmol/L.

#### 2.3.5. Antioxidant Status Parameters

GSH and GSSG levels were detected using a spectrofluorophotometer (Fluorate 02-ABFF-T, Bioanalytics, Saint Petersburg, Russia), with tests performed at λ = 350 nm (excitation) and λ = 420 nm (emission). GSH reacts specifically with o-phthalaldehyde at pH = 8.0 to form a fluorescent product that can be activated at 350 nm with an emission peak at 420 nm. GSSG determination was carried out similarly with o-phthalaldehyde, but in a more alkaline environment (pH = 12.0). In addition, N-ethylmaleimide was added to the samples to prevent the oxidation of GSH to GSSG. The fluorescence recording conditions were identical in both tests [30]. Concentrations are expressed in mmol/L.

GR and GP activities were assayed using commercial kits (Randox Laboratories Ltd., Crumlin, UK; GR2368, RS504; BioChemMack distributor) on an automatic photometer (BTS-330, BioSystems, Barcelona, Spain). GR catalyzes the reduction of GSH in the presence of NADPH, which is oxidized to NADP+. Using cumene hydroperoxide, GP catalyzes the oxidation of glutathione, which is then immediately reduced with the oxidation of NADPH to NADP+ in the presence of GR and NADPH. The results were calculated according to the manufacturer’s recommendations. The enzyme concentration required to catalyze the conversion of 1.0 μmol of substrate per min at 37 °C was taken as the unit of enzyme activity. Absorbance changes were measured at λ = 340 nm for GR at intervals of 1 min for 5 min and for GP, at intervals of 1 min for 3 min. Enzyme activity is expressed in units per 1 L of serum (for GR) or hemolysate (for GP) (U/L).

GSTpi concentrations (ng/mL of serum) were determined using commercial enzyme-linked immunosorbent assay kits (Cloud-Clone Corporation, Houston, TX, USA; SEB090Hu; BioChemMack distributor) on a microplate reader (MultiSkan ELX808, Biotek, Winooski, VT, USA) at λ = 450 nm.

The SOD activity in erythrocytes was determined using commercial kits (RANSOD, Randox Laboratories Ltd., Crumlin, UK; SD125; BioChemMack distributor) and a spectrofluorophotometer (BTS-350, BioSystems, Barcelona, Spain) in accordance with the manufacturer’s instructions. The SOD method involves the use of xanthine and xanthine oxidase to generate superoxide radicals that react with 2-(4-iodophenyl)-3-(4-nitrophenyl)-5 (phenyl) tetrazolium chloride to form a red formazan dye. The enzyme activities are expressed in equivalent units.

The TAS was determined using commercial kits (Randox Laboratories Ltd., Crumlin, UK; NX2332; BioChemMack distributor). In brief, 2,2′-azino-bis (3-ethylbenzothiazoline-6-sulfonic acid) (ABTS) was incubated with peroxidase (metmyoglobulin) and H_2_O_2_ to produce the radical cation ABTS. The measurements were carried out using a spectrofluorophotometer (BTS-350, Barcelona, Spain) at λ = 600 nm. The TAS is expressed in conventional units.

#### 2.3.6. Statistical Analysis

The obtained data were processed using STATISTICA 10 (Stat-Soft Inc., Tulsa, OK, USA). A power calculation was not performed for this analysis. The visual graphic method and the Kolmogorov–Smirnov test were used to determine the proximity to the normal law of distribution of quantitative signs. Fisher’s test was used to determine the equality of the general variances. Data related to age and BMI (normal distribution) are presented as the arithmetic mean ± standard deviation and were analyzed using the parametric Student’s *t*-test. The endothelin isoforms, oxidative stress, and antioxidant status parameters (non-normal distribution) are presented as the median (quartile 1 (Q1); quartile 3 (Q3)). The analysis of intergroup differences for independent samples was carried out using the Kruskal−Wallis ANOVA via a ranks and Median test, followed by a post-hoc (multiple comparisons) analysis using the Mann−Whitney *U*-test. Intragroup differences were determined using the Wilcoxon W-test. A test for detecting statistical outliers was carried out. The diagnostic value, optimal cut-off levels, area under curve (AUC), 95% confidence interval (CI), sensitivity, and specificity of the studied parameters were determined based on a ROC analysis. The correlation analysis was estimated using the Spearman correlation. All differences were considered statistically significant at *p* < 0.05.

## 3. Results

### 3.1. Basic Characteristics of the Groups

The basic characteristics of the groups are given in Table 1. Higher levels of neutrophils, lymphocytes, monocytes, and CRP and lower levels or the absence of eosinophils were detected in the patients with COVID-19+ as compared to the control group and those with asymptomatic COVID-19 (*p* < 0.05). Thrombocyte and hemoglobin levels were decreased in COVID-19+ patients as compared to those in the asymptomatic COVID-19 group (*p* < 0.05).

Moreover, we found an increased incidence of hypertension in patients with moderate COVID-19 compared to the control group. In 12.5% of the women who agreed to undergo an examination in the post COVID-19 period, an increase in blood pressure was first registered, which did not stabilize on its own after the disease. In 31.25% of women with hypertension and in 56.25% of patients without hypertension, no significant changes in blood pressure levels were found 12 months after the disease. It is worth noting that, both in the acute phase and in the post COVID-19 period, patients with hypertension required the introduction of additional medications to correct their blood pressure.

### 3.2. Endothelin Isoforms, Oxidative Stress, and Antioxidant Status Parameters in the Control Group and in COVID-19 Patients

Table 2 summarizes the endothelin isoforms, oxidative stress, and antioxidant status parameters in menopausal women with COVID-19 and in the control group. Patients with COVID-19+ had lower GPx (*p* = 0.021) and SOD (*p* = 0.002) activities and AGEs’ (*p* = 0.040) concentrations and higher GR activity (*p* = 0.0007) and GSTpi (*p* = 0.0001), TBARs (*p* = 0.010), and END-1 (*p* = 0.005) and END-2 (*p* = 0.00001) concentrations compared to those in the control group.

The 8-OHdG (*p* = 0.024) and GSSG (*p* = 0.041) levels and TAS (*p* = 0.049) were lower while the GSH (*p* = 0.049) level and GSH/GSSG ratio (*p* = 0.001) were higher in the group with asymptomatic COVID-19 as compared to the control group.

Additionally, there were differences between COVID-19+ patients and the group with asymptomatic COVID-19. The patients with COVID-19+ had lower GPx (*p* = 0.001) and SOD (*p* = 0.003) activities a lower AGEs (*p* = 0.007) concentration, a higher TAS (*p* = 0.017), and greater GR activity (*p* = 0.000006) and GSTpi (*p* = 0.00001) and TBARs (*p* = 0.034) concentrations as compared to those in the group without clinical symptoms.

### 3.3. Receiver Operator Characteristic (ROC) Analysis

In this study, an ROC analysis was carried out to determine the discriminatory abilities of endothelin isoforms, oxidative stress, and antioxidant system parameters in the diagnosis of patients with asymptomatic COVID-19 and moderate COVID-19. For the ROC analysis, all indicators were examined in order to select the most significant ones. The usefulness of the studied biomarkers in asymptomatic COVID-19 versus the control group and patients with COVID-19+ versus those with asymptomatic COVID-19 are presented in Table 3 and Table 4, respectively. 

**Figure 2:**
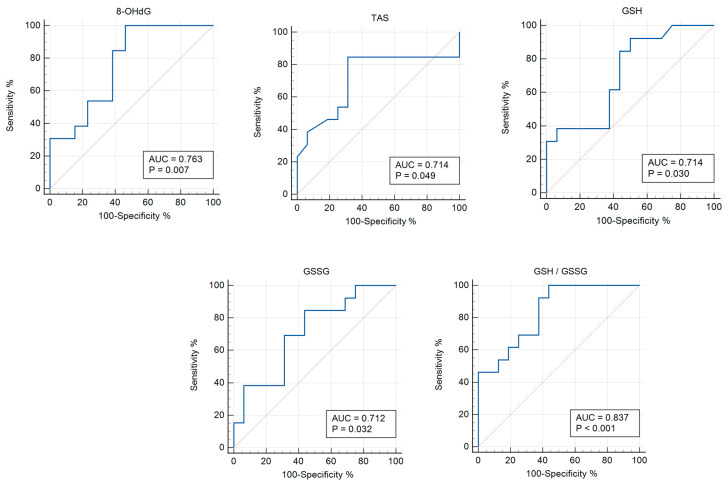
ROC curves (blue lines) of the studied biomarkers in women with asymptomatic COVID-19 versus the control group (*p* ≤ 0.05). Red lines are ROC curves of random algorithm.

**Figure 3:**
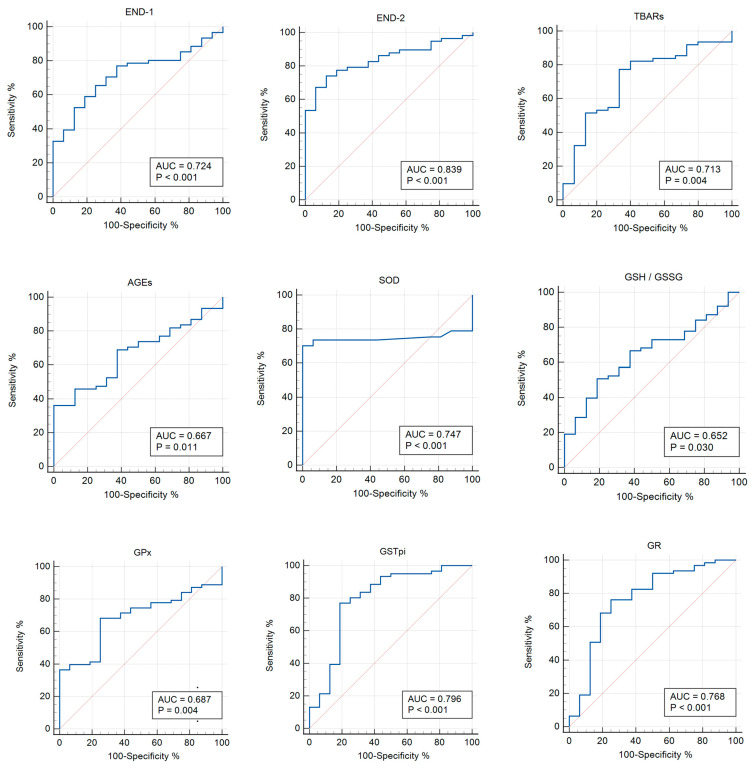
ROC curves (blue lines) of the studied biomarkers in women with COVID-19+ versus the control group (*p* ≤ 0.05). Red lines are ROC curves of random algorithm.

The ROC analysis shows the diagnostic significance of 8-OHdG (AUC 0.763; *p* = 0.006), TAS (AUC 0.714; *p* = 0.048), GSH (AUC 0.714; *p* = 0.030), GSSG (AUC 0.712; *p* = 0.031), and GSH/GSSG (AUC 0.837; *p* < 0.0001) for the group with asymptomatic COVID-19 versus the control group (Table 3; Figure 2). In addition, the significance of the parameters was identified for END-1, END-2, TBARs (AUC 0.713; *p* = 0.004), AGEs (AUC 0.667; *p* = 0.011), SOD (AUC 0.747; *p* < 0.001), GSH/GSSG (AUC 0.652; *p* = 0.030), GPx (AUC 0.687; *p* = 0.004), GSTpi (AUC 0.796; *p* < 0.001), and GR (AUC 0.768; *p* < 0.001) for the group with COVID-19+ versus the control (Table 4, Figure 3).

**Figure 4:**
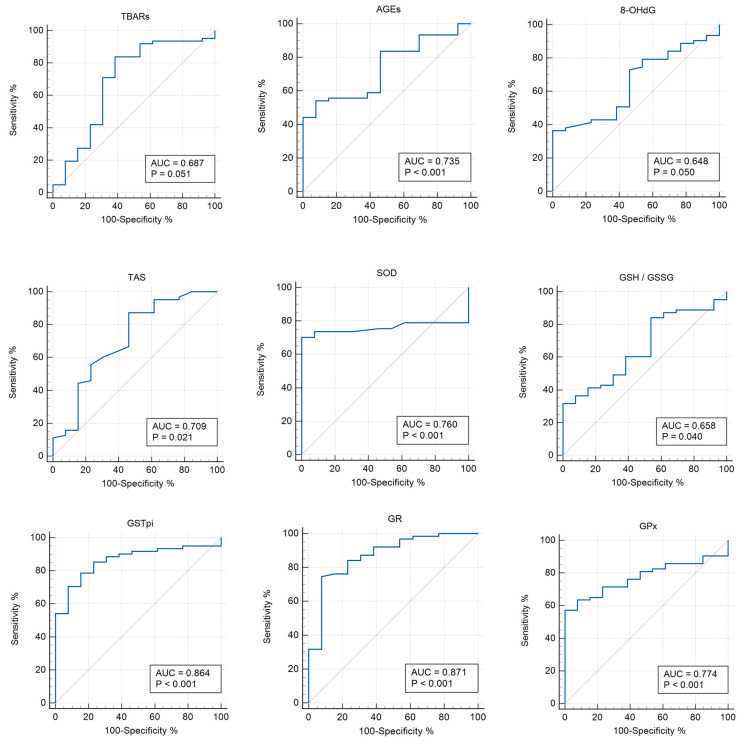
ROC curves (blue lines) of the studied biomarkers in women with COVID-19+ versus those with asymptomatic COVID-19 (*p* < 0.05). Red lines are ROC curves of random algorithm.

When comparing the groups with COVID-19+ and asymptomatic COVID-19, the significance of the parameters was identified for TBARs (AUC 0.687; *p* = 0.051), AGEs (AUC 0.735; *p* = 0.0004), 8-OHdG (AUC 0.648; *p* = 0.050), TAS (AUC 0.709; *p* = 0.020), SOD (AUC 0.760; *p* < 0.0001), GSH/GSSG (AUC 0.658; *p* = 0.039), GPx (AUC 0.774; *p* < 0.0001), GSTpi (AUC 0.864; *p* < 0.001), and GR (AUC 0.871; *p* < 0.0001) (Table 5, Figure 4).

### 3.4. Correlation Analysis

Correlations between endothelin isoforms, oxidative stress, and antioxidant system parameters in menopausal women with COVID-19 and the control group are presented in Table 6. In the control group, correlations were found between glutathione system parameters and END-2, -3, oxidative damage proteins with DNA.

In the asymptomatic group, negative correlations were observed between GSH and GSH/GSSG and lipid peroxidation products and END-2. Additionally, in this group, positive correlations were found between the products of oxidative damage to lipids and proteins and endothelin isoforms, as well as between TAS and 8-OHdG.

In the group with COVID-19+, all correlations were weak. The relationships between AGEs and GSH, GSH/GSSG, and END-2 were reversed compared to those for the control group.

### 3.5. Endothelin Isoforms, Oxidative Stress, and Antioxidant Status Parameters 12 Months Post COVID-19+

**Figure 5:**
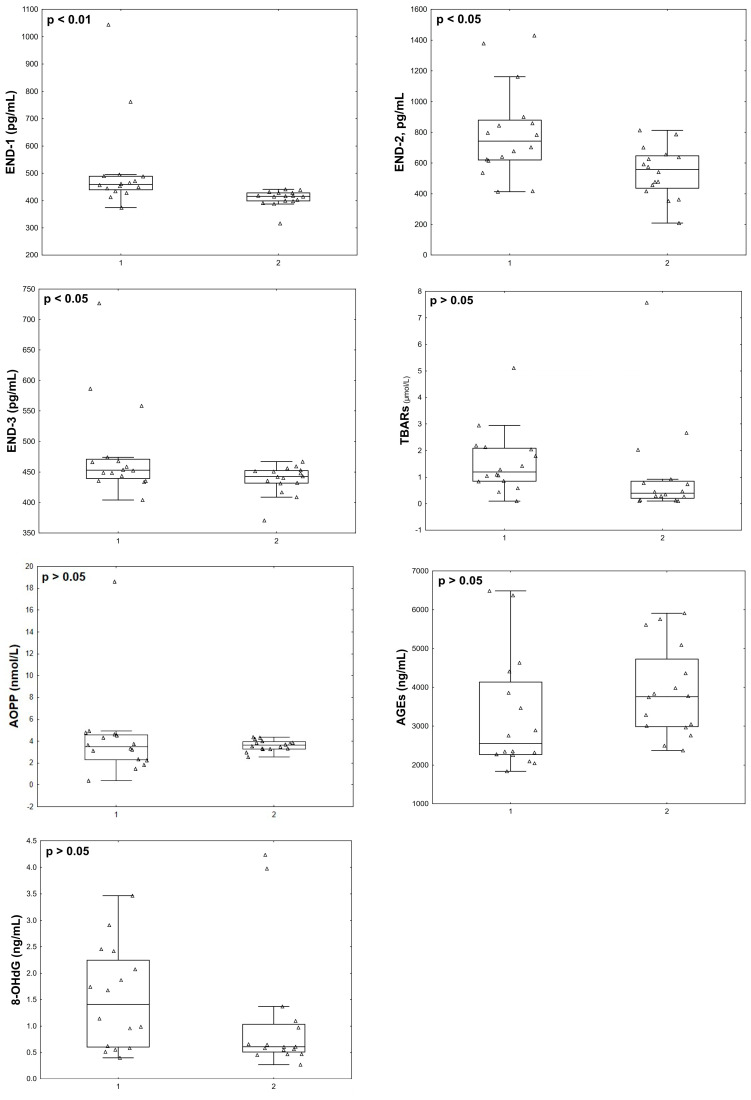
Endothelin isoforms and oxidative stress parameters in menopausal women with COVID-19 (group 1, *n* = 16) and 12 months after infection with COVID-19 (group 2, *n* = 16).

**Figure 6:**
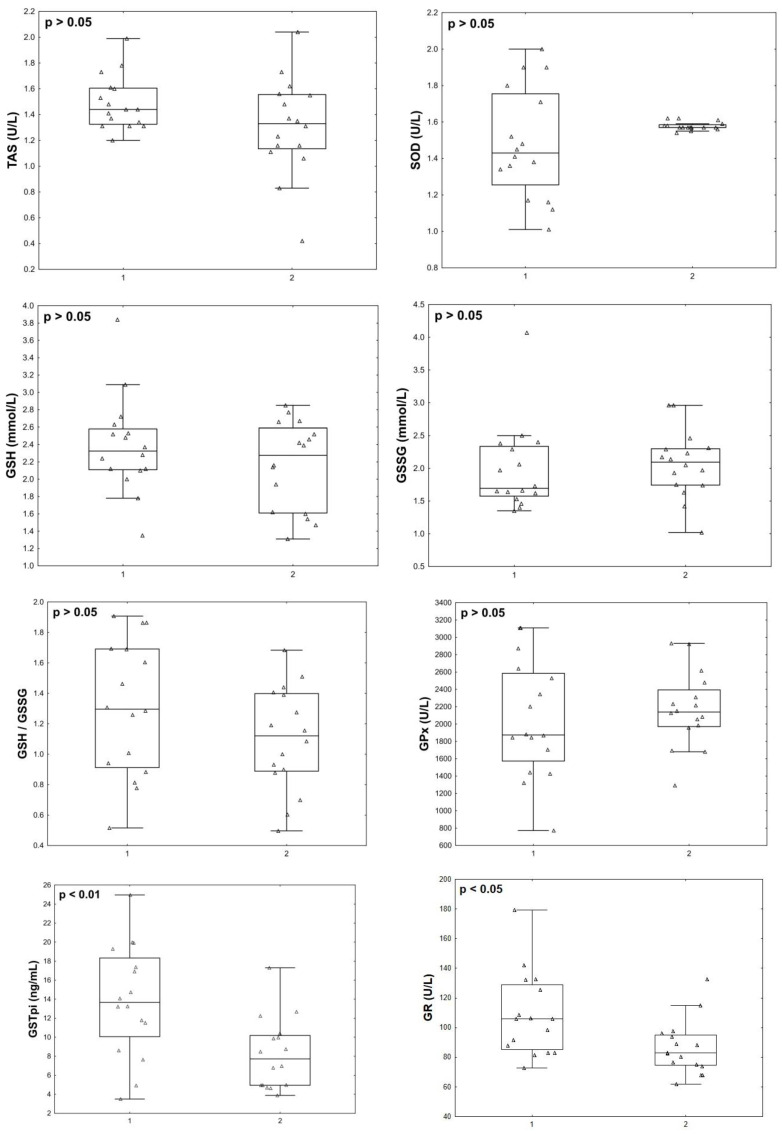
Antioxidant status parameters in menopausal women with COVID-19 (group 1, *n* = 16) and 12 months after infection with COVID-19 (group 2, *n* = 16).

At the final stage, we compared the parameters studied in the post COVID-19+ group with the data of the same women, but in the acute phase. Twelve months after infection with COVID-19+, the patients’ END-1 (*p* = 0.002), END-2 (*p* = 0.012), END-3 (*p* = 0.029), GSTpi (*p* = 0.008) concentrations, and GR activity (*p* = 0.043) decreased compared to the levels observed during the acute phase of the disease (Figure 5 and Figure 6).

## 4. Discussion

This investigation presents the first assessment of endothelin isoforms, oxidative stress, and antioxidant status in menopausal women with varying levels of COVID-19 infection. During infection with COVID-19, hypoxia, as a result of pneumonia, intensifies lipid peroxidation processes, leading to the development of oxidative stress and endothelium damage. Subsequently, the vasoconstrictive compounds END-1 and END-2 are released. Damage to the endothelial cells is also associated with damage to various organs. Accordingly, Varga et al. (2020) found fragments of the virus in the endothelium of the microvasculature of the lungs, heart, kidneys, liver, and small intestine, which explains the increase in not only END-1 but also END-2 levels [31].

Menopause is a risk factor for the development of oxidative and carbonyl stress [32,33]. Changes in the antioxidant status of the blood serum and in the cells of various organs have been associated with age-related estrogen deficiency [34,35,36,37] and respiratory viral infections [38,39]. Accordingly, we measured various markers of antioxidant status and oxidative stress in the menopausal women included in the current study. We found a decrease in SOD activity in women with moderate COVID-19. Other studies have shown inconsistent changes in SOD activity in patients with COVID-19, with both decreasing [26] and increasing [14] values being observed. Decreased SOD activity has been observed in severe and critical COVID-19 cases compared with patients with mild disease [26]. Additionally, an increase in enzyme activity regardless of the severity of the disease was detected in a study conducted on elderly patients [14]. However, the results of a study of patient groups that accounted for gender and age showed that women with COVID-19 aged over 36 years have lower SOD activity than those aged 18–35 years. This is likely because there is an age-dependent decrease in estrogen levels in the female body, which can change the enzymatic activity of the AOD system and SOD. Accordingly, a decrease in SOD mRNA expression and a simultaneous decrease in estrogen levels were detected in women with surgical menopause; however, after hormone replacement therapy, these indicators increased [40].

GSH production may also decrease in response to COVID-19 infection. This may be due to increased levels of interleukin-6 and transforming growth factor-β, the intracellular generation of free radicals, and the inhibition of BRCA1 [41]. The results of a glutathione status study showed lower GSH levels in patients with COVID-19 compared to reference values [15] and a control group [16]; levels did not differ between groups of patients in non-intensive and intensive care units [13]. There were no differences found in the GSH and GSSG levels and no differences in their ratio or in GST activity between groups with moderate and severe disease [12]. However, investigations have demonstrated differences in the GSH level in COVID-19 patients based on the severity of the disease [24,25]. In our study, we showed that glutathione system enzymes are activated in patients with moderate COVID-19. These patients exhibit lower GPx activity and, simultaneously, higher GR activity and GSTpi levels. This is likely a response to the excessive formation of highly toxic lipid peroxidation products, which are formed due to hypoxia during pneumonia. It has been shown that glutathione levels change during lung inflammation [42]. Considering that GPx kinetics are influenced by the GSH level [22], this decrease in enzyme activity is most likely due to a decrease in the GSH level, according to the principle of direct feedback. Al-Hakeim et al. (2023) showed that a decreased SpO_2_ level during the acute phase of COVID-19 significantly predicts decreased GPx activity in cases of long COVID [43].

In addition, it is known that GPx activity can be induced by the hormone melatonin [44]. Serum melatonin levels are reduced in patients with COVID-19 due to the disruption of its synthesis pathway by SARS-CoV-2 [45,46]. This may also be the reason for the decreased GPx activity detected in COVID-19 patients in our study. Higher GSTpi levels, as seen in our symptomatic COVID-19 group, are required for timely detoxification processes, as they catalyze the reactions of GSH conjugations with reactive oxygen and free radical oxidation products. Given the possibility of increased GSH consumption, timely GSSG restoration is necessary. This occurs when GR activity is increased, as we observed in our study. Considering the control levels of both GSH and GSSG in women with symptomatic COVID-19, it can be assumed that the activity of these enzymes is sufficient to maintain thiol–disulfide equilibrium. However, an increased TBARs level in this group of patients may indicate insufficient activity of the glutathione system. We identified various correlations between the parameters of the glutathione system with the oxidation products of biosubstrates and ENDs during the acute phase of the disease. Particularly notable is the change in the direction of the relationship between GSH and AGEs in COVID-19 cases compared to in the control group, which may indicate a disruption in the mechanisms of the interaction between the components of antioxidant defense and free radical oxidation processes.

At the same time, the reduced AGEs level in patients with moderate COVID-19 is noteworthy. We consider several possible reasons for this. First, against the background of intoxication syndrome in patients with pneumonia, the intake of proteins from food may be reduced, which accordingly reduces their levels of glycation products. Second, increased proinflammatory cytokine levels may contribute to a more active proteolysis process [47]. As a result, the protein level in the body may decrease, and RAGE receptors, as protein molecules, may also be damaged. It is RAGE that recognizes and binds AGEs as one of its ligands. The result of this interaction is the translocation of nuclear factor-κB into the cell nucleus and the initiation of transcription for various protein genes, including RAGE [48]. In this way, the principle of positive feedback works; that is, a decreased RAGE content leads to a decrease in the AGEs level. On the contrary, it is possible that more active binding of RAGE to AGEs occurs, since RAGE is known to activate the CD147 protein, which may be a direct receptor for SARS-CoV-2 [49]. In this case, the RAGE–AGE complex leads to the development of inflammation in the alveolar epithelial cells of the pulmonary system with the development of pneumonia [50,51]. In addition, it has been shown that the SARS-CoV-2 virus can cause epigenetic changes in internal organs [52], which can lead to changes in the expression of various proteins, including RAGE. In this study, when comparing a group of patients in the acute phase of COVID-19 with a group of asymptomatic COVID-19 patients, based on the ROC analysis, the informativeness of 8-OHdG was found to be higher in the group with a moderate case of the disease. This indicates oxidative DNA damage in patients with clinical disease and a deficiency or defect in the cellular DNA repair system. In turn, oxidized guanine can play a regulatory role in the expression of various genes [53] and take part in the production of ENDs, as indicated by positive correlations between 8-OHdG and END-1,-2 in women with COVID-19.

It is interesting to note that the asymptomatic COVID-19 patients had higher GSH and GSH/GSSG levels than controls without any changes in enzymatic activity. It is possible that the glutathione level was associated with improved resistance in response to SARS-CoV-2 virus infection, which prevented deterioration in the functioning of organs and systems and the manifestation of clinical symptoms. Our results confirm the hypothesis of Polonikov (2020), who suggested that a higher initial GSH level is linked to milder COVID-19 symptoms [54]. Moreover, GSH has inhibitory effects on the activity of angiotensin-converting enzyme (ACE-2) and has the ability to decrease the production of reactive oxygen species via the inhibition of ACE-2, leading to decreased nuclear factor-κB signaling and providing an avenue for decreased inflammation in SARS-CoV-2-infected cells [55]. For this reason, there is no accumulation of free radical oxidation products, which can react with guanosine bases and thereby increase 8-OHdG level. Among the many markers of DNA oxidative damage, 8-OHdG is one of the most studied and frequently detected compounds in nuclear and mitochondrial DNA [56]. 8-OHdG has the ability to bind hydrogen to adenine, which ultimately leads to changes at the level of the cell genome through GC → TA transversion [57]. This type of mutation is common in the development of cancer, metabolic disorders, and also accumulates with age [58,59]. The content of 8-OHdG, which is a DNA oxidative modification product, is lower in the group with asymptomatic COVID-19 than in the control, which may indicate effective functioning of the DNA repair system in these patients. All of these factors could be reasons for the absence of clinical symptoms in patients with higher GSH levels. The revealed negative correlations between glutathione and lipid peroxidation products, as well as with END-2, can be considered as one of the confirmations of this. In addition, the direct relationship of END-2 with GSTpi may indicate the timely activation of the enzyme in response to any increase in vasoconstrictor production. It is interesting to note the positive relationships between the various END isoform levels and the biosubstrate oxidation products in this group of patients. Thus, the levels of both END-1 and END-2 are inter-related with the products of the oxidized carbohydrates with proteins, lipids, and nucleic acid reactions, and END-2 is also interconnected with the end products of lipid peroxidation. At the same time, the END-3 level correlates only with the products of proteins’ oxidative modification and has a relationship with END-1. This may indicate the different mechanisms of the relationship between the production of endothelin isoforms and free radical oxidation processes. We also identified a lower TAS level in this group compared to those in both the control group and the group with moderate COVID-19. According to the studies conducted, there is no clear consensus on this parameter. Similarly, Esmaeili-Nadimi et al. (2023) did not find any significant differences between groups with mild, moderate, and severe disease [60], nor were there differences compared with controls [61]. Meanwhile, the results of another study indicate a decreased TAS in patients in intensive care units [62]. Our study did not identify any differences in this indicator between moderate COVID-19 patients and the control group. The fact that the indicator was lower in the group with asymptomatic disease may be because, in these patients, the functioning of the AOD system occurs at a sufficient level and its activation is not required. We suggest that the identified direct correlation between TAS and 8-OHdG in this group confirms this hypothesis. The results of an earlier study that assessed quality of life indicators in patients without clinical symptoms of COVID-19 showed no differences compared with the controls; however, scores for physical condition were higher in asymptomatic patients. Additionally, elevated high-density lipoprotein cholesterol levels were noted, as compared to the control values [7].

These results and the data assessed in the present study suggest the presence of a more advanced antioxidant system, better functioning of organs and systems, and higher levels of immune system adaption to respiratory virus infection in patients with asymptomatic COVID-19 s. During infections, hidden regulatory and damaging mechanisms are “exposed”, determining the body’s resistance to an emergency factors action. This may explain why not all people infected with COVID-19 experience symptoms. An earlier study demonstrated lower estrogen levels in a group of women with COVID-19 compared to controls [63], suggesting that hormones affect the entry of the SARS-CoV-2 virus into cells. It has also been shown that women aged 60 years and older who are receiving hormone replacement therapy are 46% less likely to test positive for SARS-CoV-2 [1]. Thus, estrogen deficiency may impair free radical homeostasis during a SARS-CoV-2 infection.

**Figure 7:**
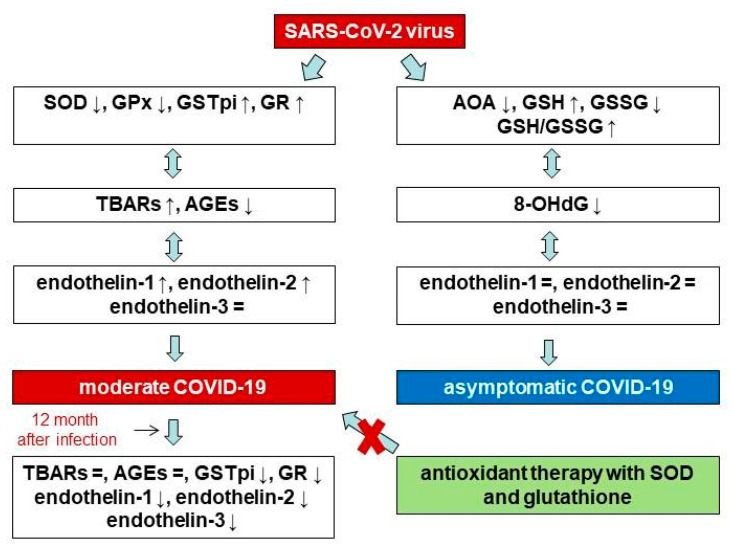
Scheme of changes in antioxidant status, oxidative stress parameters, and endothelin isoforms with different severity of COVID-19 and in the post COVID-19+ period in menopausal women.

Twelve months after patients contracted the disease, they exhibited lower GSTpi levels and GR activity as compared to during the acute phase of COVID-19. However, we did not find differences between the levels of products of biosubstrate oxidative damage during the acute phase of COVID-19 versus 12 months post-disease. Additionally, we detected decreases in not only END-1 and END-2, but also END-3, although the concentration of END-3 during the acute phase of the disease did not differ from the values in the control group. Given that END-3 is found in high concentrations in nervous tissue, our results may be further evidence of the disruption of the nervous system during infection with the SARS-CoV-2 virus. This may be because the levels of reactive oxygen species and free radical oxidation products decrease. In fact, Hofmann et al. (2023) measured the post-COVID-19 blood superoxide anion levels in hospital employees who were suffering from fatigue-like symptoms. They compared groups from, on average, three months after recovery from COVID-19 and eight weeks thereafter. It was shown that decreases in superoxide anion and oxidative stress-induced DNA strand breaks coincided with the attenuation of fatigue symptoms [64]. A study conducted by Stufano et al. (2023) showed that, in non-hospitalized patients, four months after testing negative for COVID-19, malondialdehyde levels were higher than in a control group, but hydrogen peroxide levels did not differ between groups [65]. Hypoxia and acute stress are known to be inducers of END production, during which there is an increase in the level of reactive oxygen species. In turn, by activating the nuclear factor-κB pathway, reactive oxygen species contribute to an increase in pro-inflammatory cytokines, and macrophages, neutrophils and endothelial cells are activated by NADPH oxidase, which promotes the formation and release of ENDs [3,66]. On the other hand, increased END levels can lead to increased hydrogen peroxide levels and the decreased expression of the eNOS protein [67,68]. Thus, taking into account the results of other studies [64,65], it can be assumed that the decrease in END levels in our patients in the post-COVID-19 period is associated with a decrease in the levels of reactive oxygen species in the long term, and vice versa. Scheme of changes in antioxidant status, oxidative stress parameters, and endothelin isoforms with different severity of COVID-19 and in the post COVID-19+ period in menopausal women are presented in Figure 7.

This study has some limitations. First, only a small number of patients agreed to undergo a further examination 12 months after contracting COVID-19. However, the inclusion of the same women in the study (as patients and 12 months after contracting the disease) eliminates this limitation. Second, the study was limited by the fact that a small number of women were assigned to the control group due to the high prevalence of the SARS-CoV-2 virus. Also, the absence of hormones and their relationships with the studied parameters is a limitation of this study.

## 5. Conclusions

The results of the current study may indicate a decreased activity of the primary link in the AOD system (SOD, GPx) and insufficient activation of the enzymatic link in the glutathione system (GSTpi, GR), leading to the overproduction of reactive oxygen species and, as a consequence, excessive formation of ENDs in patients with moderate COVID-19. Further, the high content of lipid peroxidation products 12 months post-disease, despite decreases in the END concentration and the activity of enzymes that catalyze glutathione conjugation with nonpolar substrates and its further restoration, indicates long-term changes in the homeostasis of free radicals. These results do not exclude the possibility of endothelial dysfunction in the long-term post-COVID-19 period, since we only examined the END levels, although there are many markers of endothelial dysfunction. Asymptomatic patients with COVID-19 exhibit increased levels of glutathione, which may improve resistance to infection with the SARS-CoV-2 virus, not only preventing the manifestation of clinical symptoms but also contributing to the effective functioning of the DNA repair system. The results of the current study suggest that particular attention should be paid to menopausal women who contract COVID-19 as they may be more vulnerable to the negative effects of this disease.

## Figures and Tables

**Table 1:** Profile of groups.

Parameters	Control	Asymptomatic COVID-19	COVID-19+	*P_(_*_ANOVA)_	*P*χ^2^
	*n* = 16	*n* = 13	*n* = 63		
Age, years	57 ± 6.24	54 ± 7.75	58 ± 6.4	0.175	-
BMI, kg/m^2^	27.04 ± 3.69	28.63 ± 4.96	30.03 ± 5.96	0.143	-
Systolic blood pressure, mmHg	127.6 ± 11.27	126.15 ± 16.6	111.38 ± 9.23	0.015	-
Diastolic blood pressure, mmHg	78.7 ± 8.34	77.69 ± 5.63	66.74 ± 4.39	0.203	-
Heart rate, bpm	69.5 ± 3.58	71.6 ± 4.87	93.7 ± 4.12	0.011	-
Type 2 diabetes mellitus, %	-	-	15.9	-	0.076
Hypertension, %	25	38.5	66.7	-	0.005
Kidney disease, %	-	15.4	4.76	-	0.176
Thyroid disease, %	12.5	7.69	9.52	-	0.904
Erythrocytes, 10^12^/L	4.4 (4.27; 4.58)	4.73 (4.46; 4.81)	4.58 (4.2; 4.87)	0.081	-
Eosinophils, 10^9^/L	0.1 (0.08; 0.15)	0.09 (0.06; 0.12)	0 (0; 0.1) *, ^	0.001	-
Thrombocytes, 10^9^/L	251 (217; 280)	278 (226.25; 316)	220 (171; 269) ^	0.083	-
Hemoglobin, g/L	135 (128; 144)	141.5 (138; 145.5)	134 (125; 143) ^	0.045	-
CRP, mg/L	4.25 (2.7; 8.8)	2.2 (1; 6.3)	12 (6.8; 13.5) *, ^	0.0006	-
ALT, U/L	23.2 (19.05; 29.4)	21.35 (18; 29.5)	30 (20; 46.8)	0.141	-
AST, U/L	27.7 (27.4; 31.1)	27.1 (24.3; 32.7)	36.1 (27; 46)	0.104	-
Glucose, mM/L	5.08 (4.22; 5.46)	4.97 (4.48; 5.37)	7.23 (5.9; 9.11)	0.000	-

*, *p* < 0.05 compared to the control; ^, *p* < 0.05 compared to asymptomatic COVID-19.

**Table 2:** Endothelin isoforms, oxidative stress, and antioxidant status parameters in menopausal women with COVID-19, asymptomatic COVID-19, and in the control group.

Parameters	Control	Asymptomatic COVID-19	COVID-19+	*P*_(ANOVA)_
	*n* = 16	*n* = 13	*n* = 63	
END-1, pg/mL	408.89 (377.68; 431.55)	445.57 (406.77; 455.88)	449.62 (414.53; 496.45) *	0.047
END-2, pg/mL	479.67 (423.99; 520.80)	557.22 (463.85; 711.01)	673.69 (536.16; 843.8) *	0.001
END-3, pg/mL	434.79 (380.13; 470.31)	440.61 (422.26; 449.12)	443.46 (406.66; 489.59)	0.33
TBARs, μmol/L	0.65 (0.35; 1.17)	0.47 (0.27; 1.33)	1.28 (0.82; 1.8) *, ^	0.175
AOPP, nmol/L	3.62 (3.27; 4.15)	3.76 (3.48; 4.15)	3.76 (2.38; 4.62)	0.61
AGEs, ng/mL	3813.52 (2633.73; 4588.35)	4691.18 (2932; 6072.88)	2755.78 (2318.88; 3990.91) *, ^	0.287
8-OHdG, ng/mL	1.40 (0.62; 1.74)	0.55 (0.46; 1.06) *	0.92 (0.54; 1.68)	0.09
TAS, U/L	1.48 (1.29; 1.55)	1.27 (1.11; 1.44) *	1.45 (1.34; 1.6) ^	0.007
SOD, U/L	1.58 (1.55; 1.58)	1.59 (1.57; 1.62)	1.24 (0.92; 1.59) *, ^	0.001
GSH, mmol/L	2.02 (1.7; 2.52)	2.45 (2.26; 3.08) *	2.35 (2.07; 2.73)	0.046
GSSG, mmol/L	2.1 (1.82; 2.37)	1.84 (1.62; 1.95) *	1.87 (1.62; 2.34)	0.072
GSH/GSSG	0.93 (0.85; 1.22)	1.44 (1.16; 1.78) *	1.26 (0.89; 1.51)	0.016
GPx, U/L	2126 (1820.5; 2412.5)	2377 (2056; 2558)	1804 (1321; 2162) *, ^	0.002
GSTpi, ng/mL	5.01 (3.67; 10.59)	6.02 (4.94; 7.85)	14.15(11.52; 18.2) *, ^	0.000
GR, U/L	79.3 (70.75; 86.65)	73.3 (63.3; 79.6)	101.4 (86.1; 115.4) *, ^	0.00001

*, *p*(U) < 0.05 compared to the control; ^, *p*(U) < 0.05 compared to the group with asymptomatic COVID-19.

**Table 3:** ROC analysis of endothelin isoforms, oxidative stress, and antioxidant system parameters in women with asymptomatic COVID-19 in comparison with the control group (*p* ≤ 0.05).

Parameter	AUC	*p*-Value	Cut-Off Point	95% CI	Sensitivity	Specificity
8-OHdG	0.763	0.006	≤1.203	0.557–0.906	100	53.85
TAS	0.714	0.048	≤1.46	0.517–0.865	84.62	68.75
GSH	0.714	0.030	>1.89	0.517–0.865	92.31	50.00
GSSG	0.712	0.031	≤2.08	0.514–0.863	84.62	56.25
GSH/GSSG	0.837	<0.0001	>0.947	0.653–0.947	100	56.25

**Table 4:** ROC analysis of endothelin isoforms, oxidative stress, and antioxidant system parameters in women with COVID-19+ in comparison with the control group (*p* ≤ 0.05).

Parameter	AUC	*p*-Value	Cut-Off Point	95% CI	Sensitivity	Specificity
END-1	0.724	<0.001	>428.57	0.611–0.820	65.6	75
END-2	0.839	<0.010	>549.54	0.736–0.915	74.1	87.5
TBARs	0.713	0.004	>0.759	0.598–0.810	77.4	66.7
AGEs	0.667	0.011	≤2488.56	0.550–0.770	36.1	100
SOD	0.747	<0.001	≤1.48	0.636–0.858	70.2	100
GSH/GSSG	0.652	0.030	>1.23	0.515–0.789	50.8	81.2
GPx	0.687	0.004	≤1931	0.561–0.812	68.3	75
GSTpi	0.796	<0.001	>11.32	0.649–0.943	77	81.2
GR	0.768	<0.001	>84.9	0.620–0.916	76.2	75

**Table 5:** ROC analysis of endothelin isoforms, oxidative stress, and antioxidant system parameters in women with COVID-19+ versus those with asymptomatic COVID-19 (*p* ≤ 0.05).

Parameter	AUC	*p*-Value	Cut-Off Point	95% CI	Sensitivity	Specificity
TBARs	0.687	0.051	>0.553	0.570–0.789	83.87	61.54
AGEs	0.735	0.0004	≤2799.07	0.620–0.831	54.10	92.31
8-OHdG	0.648	0.050	>1.203	0.530–0.754	36.51	100
TAS	0.709	0.020	>1.27	0.593–0.807	87.30	53.85
SOD	0.760	<0.0001	≤1.48	0.643–0.854	70.18	100
GSH/GSSG	0.658	0.039	≤0.941	0.540–0.763	31.75	100
GPx	0.774	<0.0001	≤1833	0.664–0.862	57.14	100
GSTpi	0.864	<0.001	>10.37	0.764–0.932	78.69	84.62
GR	0.871	<0.0001	>86.1	0.775–0.937	74.60	92.31

**Table 6:** Correlations between endothelin isoforms, oxidative stress, and antioxidant status parameters in menopausal women with COVID-19, asymptomatic COVID-19, and the control group (*p* < 0.05).

	Control		Asymptomatic COVID-19		COVID-19	
Correlation	*r*	*p*	*r*	*p*	*r*	*p*
GSH/GSH/GSSG	0.82	0.000	0.64	0.019	0.61	0.00000
GSSG/GPx	0.66	0.005				
GSH/AGEs	0.61	0.013			−0.38	0.002
GSH/END-2	−0.58	0.018	−0.68	0.011		
GSSG/GSH/GSSG	−0.57	0.022			−0.73	0.00000
GSSG/8-OHdG	0.56	0.045				
GSH/GSSG/AGEs	0.63	0.009			−0.37	0.003
GPx/END-3	0.52	0.040			0.35	0.006
GSTpi/AOPP	−0.68	0.003				
AGEs/AOPP	0.58	0.018				
AGEs/END-2	−0.60	0.013	0.74	0.004		
END-1/END-3	0.68	0.004	0.55	0.049	0.67	0.00000
GSH/TBARs			−0.59	0.032		
GSH/GSSG/END-2			−0.64	0.017		
GSTpi/END-2			0.63	0.021		
SOD/AOPP			0.64	0.019		
TAS/8-OHdG			0.63	0.020		
TBARs/END-2			0.60	0.029		
AGEs/END-1			0.65	0.016		
AOPP/END-3			0.63	0.021		
GSSG/END-2					0.30	0.023
GSH/GSSG/END-3					0.31	0.017
GPx/8-OHdG					0.32	0.009
GPx/END-1					0.42	0.0006
GSTpi/8-OHdG					−0.31	0.015
8-OHdG/END-1					0.38	0.002
8-OHdG/END-3					0.32	0.014
AGEs/END-3					−0.30	0.022
GPx/SOD					0.33	0.010
SOD/8-OHdG					0.36	0.005
SOD/AGEs					−0.32	0.013
SOD/END-1					0.35	0.006
SOD/END-3					0.30	0.024
TAS/AOPP					0.36	0.003

## Data Availability

The data presented in this study are available on request from the corresponding author due to privacy.

## Abbreviations

8-OHdG	8-hydroxy-2-deoxyguanosine
ABTS	2,2′-azino-bis (3-ethylbenzothiazoline-6-sulfonic acid
AGEs	advanced glycation end products
ALT	alanine aminotransferase
AMH	anti-Mullerian hormone
AOD	antioxidant defense system
AOPP	advanced oxidation protein products
AST	aspartate aminotransferase
AUC	area under curve
BMI	body mass index
BRCA1	breast cancer 1
CI	confidence interval
CRP	C-reactive protein
DM2	type 2 diabetes mellitus
DNA	deoxyribonucleic acid
EDTA-K3	ethylenediaminetetraacetic acid
END	endothelin
GPx	glutathione peroxidase
GR	glutathione reductase
GSH	reduced glutathione
GSSG	oxidative glutathione
GSTpi	glutathione S-transferase
HT	hypertension
NADP	nicotinamide adenine dinucleotide phosphate
NADPH	nicotinamide adenine dinucleotide phosphate hydrogen
RAGE	receptor for advanced glycation end products
RNA	ribonucleic acid
ROC	receiver operator characteristic
SOD	superoxide dismutase
TAS	total antioxidant status
TBARs	thiobarbituric acid reactants
